# Characterization of a serine protease inhibitor from *Trichinella spiralis* and its participation in larval invasion of host’s intestinal epithelial cells

**DOI:** 10.1186/s13071-018-3074-3

**Published:** 2018-09-06

**Authors:** Yan Yan Song, Yao Zhang, Hua Nan Ren, Ge Ge Sun, Xin Qi, Fan Yang, Peng Jiang, Xi Zhang, Jing Cui, Zhong Quan Wang

**Affiliations:** Department of Parasitology, Medical College, Zhengzhou University, 40 Daxue Road, Zhengzhou, 450052 People’s Republic of China

**Keywords:** *Trichinella spiralis*, Serine protease inhibitor, Inhibitory activity, Trypsin, Larval invasion

## Abstract

**Background:**

*Trichinella spiralis* serine protease inhibitor (TsSPI) was identified in ES proteins of adult worms (AW), the TsSPI gene was highly expressed at enteral stage worms (AW and newborn larvae), distributed mainly in the cuticle and stichosome of this nematode. Vaccination of mice with rTsSPI exhibited a 62.2% reduction of intestinal AW and a 57.25% reduction of muscle larvae after larval challenge. The aim of this study was to investigate the biological characteristics of TsSPI and its roles in the process of *T. spiralis* invasion of host’s intestinal epithelium cells (IECs).

**Methods:**

The rTsSPI inhibition on trypsin enzymatic activity was detected by SDS-PAGE and spectrophotometry. The binding of rTsSPI with intestinal epithelium from normal mice and the primary cultured mouse intestinal epithelium cells (IECs) was examined by indirect immunofluorescent (IIF), the cellular localization of rTsSPI binding to IECs was observed by confocal microscopy. The inhibition of anti-rTsSPI serum on *T. spiralis* invasion of IECs was determined by an *in vitro* invasion assay. Anti-rTsSPI antibody cytotoxicity on the newborn larvae (NBL) was also determined.

**Results:**

The rTsSPI had the inhibitory activity against porcine trypsin. The rTsSPI specifically bound to the intestinal epithelium from normal mice and primary cultured mouse IECs, and the binding sites were located in IEC membrane and cytoplasm. Anti-rTsSPI antibodies depressed the larval invasion of IECs with a dose-dependent mode. Anti-rTsSPI antibodies also participated in the destruction of *T. spiralis* NBL *via* an ADCC-mediated manner.

**Conclusions:**

TsSPI might participate in the *T. spiralis* larval invasion of IECs and it is likely the potential vaccine target against *T. spiralis* enteral stages.

## Background

Trichinellosis is a major zoonotic parasitosis resulted from the ingestion of raw or under-cooked meat infected with the nematodes *Trichinella* spp. Three classes of vertebrates (mammals, birds and reptiles) are known to act as the hosts of *Trichinella* spp. and natural infections have been described in more than 150 mammalian species [1], and patients with trichinellosis have been recorded in 55 countries in the world [2]. *Trichinella spiralis* is the main pathogen causing human trichinellosis and the important reservoir host is domestic swine [3, 4]; porcine meat is the major infectious source of *T. spiralis* infection in humans [5]. *Trichinella* infection is not only an important public health concern, but also a serious threat to animal food safety. Development of control measures is required to block the transmission of trichinellosis among animals and from animals to humans [6].

When the contaminated meat is ingested by hosts, *T. spiralis* muscle larvae (ML) are liberated in stomach under the action of digestive enzymes, migrate to the small intestine, and develop into intestinal first-stage infective larvae (IL1) [7, 8]. The IL1 invade intestinal epithelium cells (IECs) where they develop into female/male adult worms (AW) after undergoing four molts within 31 h. About 5 days post-infection (dpi), the ovoviviparous females release the newborn larvae (NBL) that penetrate into skeletal muscles, where they encapsulate and elicit the formation of nurse cell-muscle larva complex [8]. Intestinal mucosa is the first native barrier against *Trichinella* infection and the principal interaction site between intestinal parasites and the host [9, 10]. The encapsulated ML may survive for several years in living hosts without any major harm [11], but the mechanism of immune evasion of this parasite is not fully known. Theoretically, the prevention and control of *Trichinella* infection should be to interrupt the IL1 lodge in IECs, to block larval development to AW and NBL production, to interdict NBL migration and to eliminate AW and NBL from the gut [12].

Serine protease inhibitors (serpins) is a superfamily of conserved proteins which inhibit serine protease activity and act a pivotal part in inflammation, and fibrinolysis [13]. The helminth-secreted serpins protect the parasites from host’s serine proteolysis, help the worms to invade the defensive barriers, and to escape the host’s immune attack [14]. A *T. spiralis* serpin from the ML (GenBank: AF231948) has been identified and expressed. This recombinant protein inhibits trypsin activity [15]. Two serpins from *T. spiralis* inhibited the activities of chymotrypsin and pepsin [16]. Another serpin screened from *T. spiralis* ML cDNA library was expressed, and vaccination of mice with rTs-serpin exhibited partial protection against *Trichinella* larval challenge [17].

Previous studies showed a *T. spiralis* serine protease inhibitor (TsSPI, GenBank: XP_003377380.1) identified in the *T. spiralis* AW excretory/secretory (ES) proteins by immunoproteomics with early infection sera [10, 18]. Bioinformatics analysis revealed that the complete TsSPI cDNA sequence was 1050 bp, encoded a 39.6 kDa protein of 349 amino acids. The SMART analysis showed that there was a functional domain with an active site containing the classic reactive central loop (RCL) of serine protease inhibitors. The TsSPI gene was cloned and expressed in our labarotory. Quantitative PCR (qPCR) and immunofluorescence assay revealed that the TsSPI gene was highly expressed at enteral stage worms (AW and NBL), principally located in the cuticle and stichosome of this nematode. Vaccination of mice with rTsSPI produced a 62.2% intestinal AW reduction and a 57.25% ML reduction after larval challenge [19]. The aim of this study was to investigate the biological characteristics of TsSPI and its roles in the course of *T. spiralis* invasion of host’s IECs.

## Methods

### Mice and parasites

Female BALB/c mice, 6 weeks old, were obtained from the Zhengzhou University Experimental Animal Center (Zhengzhou, China). *Trichinella spiralis* strain (ISS534) was collected from a domestic porcine in Henan Province of China. We kept this strain by passage in mice every 6 months.

### Collection of worms at various stages

Mice were infected orally with 300 *T. spiralis* larvae and the ML were acquired by artificial digestion of infected mouse carcasses at 42 days post-infection (dpi) [20, 21]. The IL1 were isolated from intestine at 6 h post-infection [22], and the AW were obtained from intestine at 6 dpi [23]. The 6 dpi female adults were cultured for 24 h at 37 °C for recovering newborn larvae (NBL) [24]. The IL1 excretory-secretory (ES) proteins were prepared [25].

### Cell culture and protein preparation

The primary IECs were prepared from fetal mouse intestines and susceptible to *T. spiralis* invasion [7]. The mouse striated muscle myoblast C2C12 was unsusceptible to the larval invasion and utilized as negative control [26]. The cells were cultivated by using Dulbecco’s modified Eagle’s medium and recovered following trypsinization. The IEC lysates were prepared with the help of grinding, sonicating and centrifuging as described [27]. The concentration of IEC and IL1 ES proteins was measured by the Bradford assay [28].

### The rTsSPI and anti-rTsSPI serum

The TsSPI gene was cloned, and the recombinant pQE-80L/TsSPI was transformed into *Escherichia coli* BL21 (DE3) (Novagen, La Jolla, CA, USA) [29]. The TsSPI was induced with 0.5 mM IPTG at 37 °C for 5 h, and then purified by using a Ni-NTA His-tag affinity kit (Novagen) in our laboratory [19]. Ten mice were subcutaneously immunized with 20 μg of the rTsSPI emulsified with complete Freund’s adjuvant, and boosted 3 times with the rTsSPI with incomplete Freund’s adjuvant at two weeks interval [30, 31]. Anti-rTsSPI immune serum were collected at two weeks following the fourth immunization, and pre-immune normal serum was used as negative control [32].

### SDS-PAGE analysis of rTsSPI activity for inhibiting trypsin

In order to observe the rTsSPI inhibiting effect on trypsin hydrolysis of BSA, 0.5 μg of trypsin and different concentration of rTsSPI (3–6 μg) and BSA (0.75–4.5 μg) was used in SDS-PAGE analysis with 5% stacking gels and 12% resolving gels, and stained with Coomassie brilliant blue R-250 [33]. rTsSPI was pre-incubated with trypsin in 20 μl of PBS for 30 min at 37 °C. Following centrifugation at 5000× *g* for 10 s, BSA was added and reacted for 1 h at 37 °C. The mixture of trypsin and BSA was used as positive control; negative control contained trypsin alone or rTsSPI only. Reactions were ceased by the addition of sample buffer contained 2% SDS and 1% β-mercaptoethanol. Samples were denatured at 100 °C for 5 min and separated on 12% gels [34, 35].

### Spectrophotometric assay of rTsSPI activity for inhibiting trypsin

The rTsSPI (0–18 μg) was firstly pre-incubated with trypsin (1.25 μg) in 20 μl of PBS for 30 min at 37 °C, subsequently added 100 μl of 0.5 mM N-Benzoyl-DL-arginine ethyl ester hydrochloride (BAEE; BBI, BBI CO., LTD, Shanghai, China). Under this reaction conditions, trypsin substrate BAEE was converted to N-Benzoyl-DL-arginine (BA) within 5 min. Catalytic substrate reaction occurs every minute, and the enzyme required for every 0.001 increase in absorbance at A253 nm is an enzyme activity unit [36]. Each sample had three replicates. The absorbance at 253 nm was measured by a spectrophotometer (Mapada, Shanghai, China). The inhibition rate was calculated as follows:

$$ \mathrm{Inhibition}\ \mathrm{rate}\ \left(\%\right)=\left[\left(\mathrm{Total}\ \mathrm{enzyme}\ \mathrm{activity}\ \mathrm{units}\hbox{-} \mathrm{Residual}\ \mathrm{enzyme}\ \mathrm{activity}\ \mathrm{units}\right)/\mathrm{Total}\ \mathrm{enzyme}\ \mathrm{activity}\ \mathrm{units}\right]\times 100 $$

### Effect of temperature and pH on stability of rTsSPI activity for inhibiting trypsin

The rTsSPI (1.5 μg/μl, 100 mM Tris-HCl, pH 8.0) was heated at 30–100 °C for 30 min, subsequently cooled at room temperature, and residual enzymatic activity of trypsin was measured. To assay the pH stability, the rTsSPI solution (1.5 μg/μl) was diluted with an equal volume of 100 mM different buffers: sodium citrate, pH 2–4; sodium acetate, pH 4.5–5.5; sodium phosphate, pH 6.0–7.0; Tris-HCl, pH 7.5–8.5; and sodium bicarbonate, pH 9.0–10.0. After being incubated in each buffer at 37 °C for 30 min, the pH was modulated to pH 8.0, and the rTsSPI inhibitory activity against trypsin was measured as described in [37].

After the above treatment, the residual inhibitory activity of the rTsSPI on trypsin was assayed using BAEE as substrate. Aliquots (1.5 μg/μl, 12 μl of rTsSPI) were mixed with a swine trypsin (5 μl, 0.25 μg/μl in 0.1 mM HCl) in 50 mM Tris-HCl, pH 8.0. The mixture was incubated at 37 °C for 30 min, and then 100 μl of 0.5 mM BAEE were added to make a final volume of 120 μl. The absorbance at 253 nm was measured as above described. All samples were tested in triplicate and the data are expressed as the mean ± standard deviation (SD) of the triplicate.

### Far Western analysis of rTsSPI binding with IECs

On Far Western analysis of protein interaction between rTsSPI and IECs, the protein samples of IECs were separated by SDS-PAGE analysis [27, 38, 39]. The gel was transferred onto the nitrocellulose membrane (Merck Millipore, Billerica, MA, USA) at 80 V for 40 min in a semi-dry transfer cell (Bio-Rad, Hercules, CA, USA) [11]. The membrane was cut and blocked with 5% skim milk in PBS-0.5% Tween 20 (PBST) for 2 h at 37 °C, subsequently incubated (37 °C, 2 h) with 20 μg/ml rTsSPI, and the IL1 ES proteins and bovine serum albumin (BSA) were used as control groups [39]. After washing, the strips were incubated (37 °C, 2 h) with 1:100 dilutions of different sera (anti-rTsSPI serum, infection serum or pre-immune serum). After washing, the strips were incubated for 1 h at 37 °C with 1:10,000 dilutions of anti-mouse IgG-HRP-conjugate, and colored by using DAB (Sigma-Aldrich, St. Louis, MO, USA) [40]. The IEC protein bands bound to rTsSPI were analyzed with the Alpha view Software (AIC, http://alphaview-sa1.software.informer.com) [41].

### Indirect immunofluorescent (IIF) analysis of rTsSPI binding with IEC and cellular localization

The IECs and C2C12 cells were grown to about 90% confluence on glass coverslips in DMEM medium in culture plates for 36 h [42]. The viable cell monolayer was incubated for 2 h at 37 °C with 20 μg/ml rTsSPI. The IL1 ES protein and PBS were utilized as a positive and negative control, respectively. After washing, the monolayer was fixed with 4% formaldehyde for 20 min. The monolayer was incubated with 1:10 dilutions of infection serum, anti-rTsSPI serum or pre-immune serum, subsequently incubated with 1:100 dilutions of anti-mouse IgG-FITC conjugate (Santa Cruz, Biotechnology, Dallas, Texas, USA) at 37 °C for 1 h. The monolayer cell nuclei were dyed with propidium iodide (PI). After being washed again, the cells were observed with the aid of fluorescent microscopy (Olympus, Tokyo, Japan) [43]. Furthermore, the cellular localization of rTsSPI within IECs was further examined with a laser scanning confocal microscopy [39].

### IIF analysis of binding of rTsSPI with intestinal epithelium

Small intestines and livers were obtained from uninfected mice, fixed in 4% formaldehyde and 3-μm sections were prepared with a microtome. The IIF was performed as reported with some modifications [39, 44]. Briefly, the tissue sections were respectively incubated with 1:10 dilution of anti-rTsSPI serum, infection serum or normal serum for 2 h at 37 °C. After washing, the sections were incubated with anti-mouse IgG-FITC conjugate (1:100; Santa Cruz, USA). The intestinal epithelium cell nuclei were stained with PI. Finally, the sections were observed using fluorescent microscopy (Olympus) [45].

### The *in vitro* larval invasion of IECs

To evaluate the inhibitive effects of anti-rTsSPI serum on the IEC invasion by *T. spiralis*, the activated ML was utilized in the invasion test. The ML were activated into the IL1 by 5% mouse bile for 2 h at 37 °C [9, 46]. Two hundred larvae were added into semisolid culture media (serum-free DMEM with 15 mM HEPES and 1.75% agarose), which were used to overlay the IEC monolayer. The media were supplemented with anti-rTsSPI serum (1:100 to 1:1600), or infection serum or pre-immune serum diluted at 1:100 [26]. After incubation at 37 °C for 2 h, the larvae penetrated into the monolayer were numbered under microscopy. The larvae invaded and migrated into the monolayer were counted as invaded larvae, whereas the larvae suspended in the media were counted as non-invaded larvae [47, 48]. Three independent tests for three groups of serum samples were carried out and three repeats were used to determine the larval invasion for each kind of serum.

### Antibody-dependent cell-mediated cytotoxicity (ADCC) assay

Anti-rTsSPI antibody cytotoxicity on *T. spiralis* NBL was determined [49, 50]. Infection serum and pre-immune serum were utilized as positive and negative control, respectively. Peritoneal exudates cells (PECs) were collected from peritoneal exudate of normal mice after intraperitoneal injection with 4.5 ml of sterile RPMI-1640 medium (Gibco, Waltham, USA). After five days, peritoneal cavity was washed with RPMI-1640. The cellular suspension was centrifuged at 1500× *g* for 10 min. The number of PECs was estimated in a cell counter. The cell suspensions contained 2.5 × 10^6^ cells/ml. Mouse sera were diluted to 1:50 to 1:800 with RPMI 1640 medium, and added to the 200 μl suspension contained 100 NBL and 1 × 10^5^ PECs. The suspension was incubated in a 96-well culture plate 5% CO_2_ at 37 °C for 72 h. Each assay was performed in triplicate. The larval viability treated by ADCC was estimated on the basis of their morphology and activity under microscope. The living NBL are mobile and show wriggling motion, while the dead worms are straight, inactive or disintegrated [45, 51]. The result was expressed as the percentage of dead NBL to the total number of NBL used for each assay.

### Statistical analysis

The data were statistically analyzed using the SPSS 17.0 software. The data were shown as the mean ± standard deviation (SD). Chi-square tests or one-way ANOVAs were utilized to analyze the differences between different groups. Spearman’s rank correlation (*r*) was used to analyze the TsSPI inhibitory activity against trypsin. *P* < 0.05 was considered as statistically significant.

## Results

### Inhibition of trypsin hydrolysis of BSA by rTsSPI

**Fig. 1:**
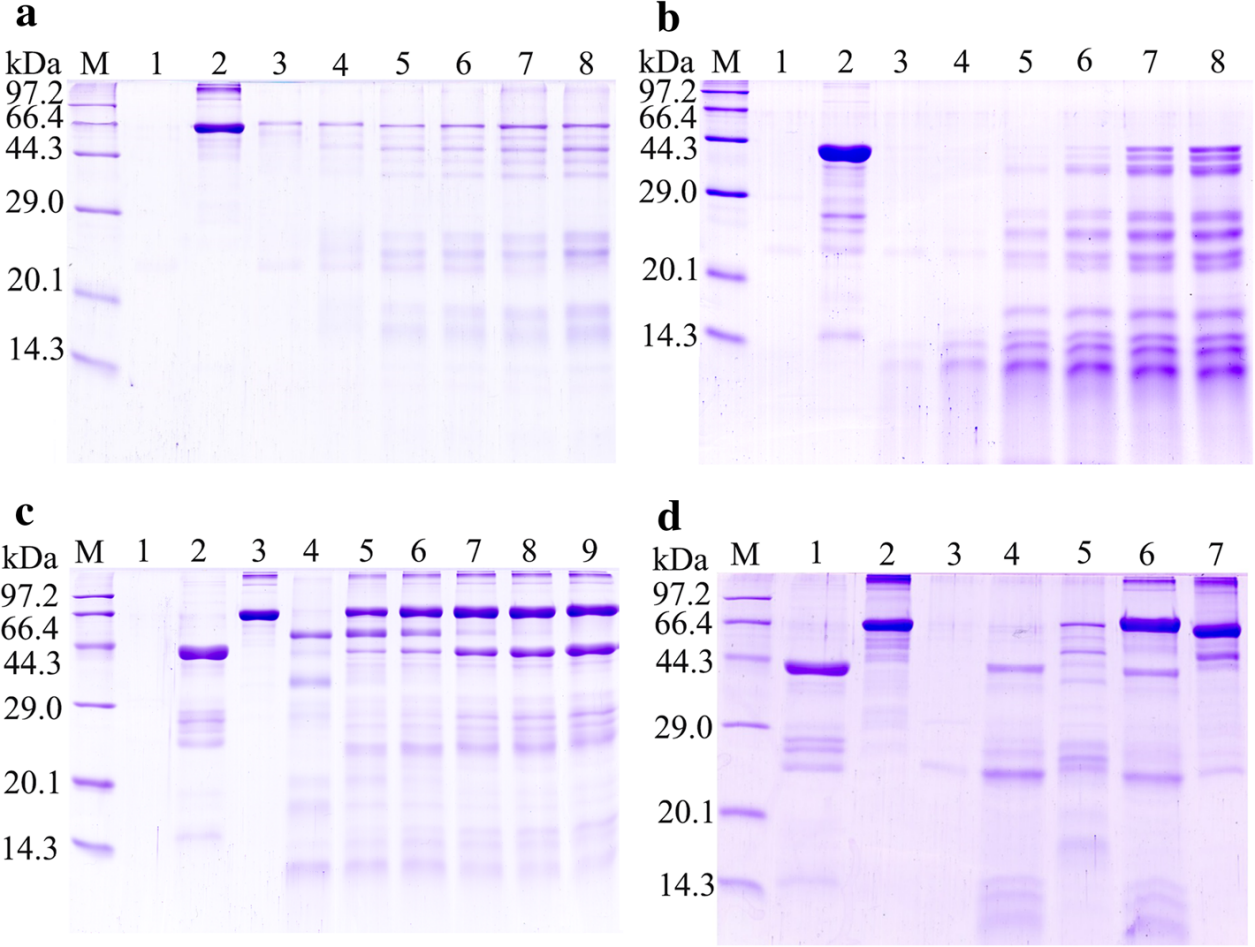
SDS-PAGE analysis of rTsSPI activity for inhibiting trypsin hydrolysis of BSA. **a** Different quantity of BSA were hydrolyzed by trypsin. Lane M: protein marker; Lane 1: trypsin; Lane 2: BSA; Lanes 3–8: trypsin + BSA (0.75, 1.50, 2.25, 3.00, 3.75 and 4.50 μg, respectively). **b** Different quantity of rTsSPI were hydrolyzed by trypsin. Lane M: protein marker; Lane 1: trypsin; Lane 2: rTsSPI; Lanes 3–8: trypsin + rTsSPI (3.00, 3.75, 4.50, 5.25, 6.00 and 6.75 μg, respectively). **c** The trypsin hydrolysis of BSA was inhibited by different quantity of rTsSPI. Lane M: protein marker; Lane 1: trypsin; Lane 2: rTsSPI; Lane 3: BSA; Lanes 4–9: trypsin + BSA + rTsSPI (3.00, 3.75, 4.50, 5.25, 6.00 and 6.75 μg, respectively). **d** The trypsin hydrolysis of BSA was inhibited by rTsSPI and natural inhibitor PMSF. Lane M: protein marker; Lane 1: TsSPI; Lane 2: BSA; Lane 3: trypsin; Lane 4: trypsin + rTsSPI; Lane 5: trypsin+BSA; Lane 6: rTsSPI+trypsin+BSA; Lane 7: PMSF+trypsin+BSA

SDS-PAGE analysis revealed that 0.50 μg trypsin could degrade different concentrations of BSA (0.75–4.50 μg) (Fig. 1a) and rTsSPI (3.00–6.75 μg) (Fig. 1b); the trypsin hydrolysis of BSA could be inhibited by rTsSPI (6.00–6.75 μg) (Fig. 1c). The proteolytic activity of trypsin was also inhibited by natural inhibitor phenylmethylsulfonyl fluoride (PMSF) (Fig. 1d).

### rTsSPI inhibitory function on trypsin activity determined by spectrophotometry

**Fig. 2:**
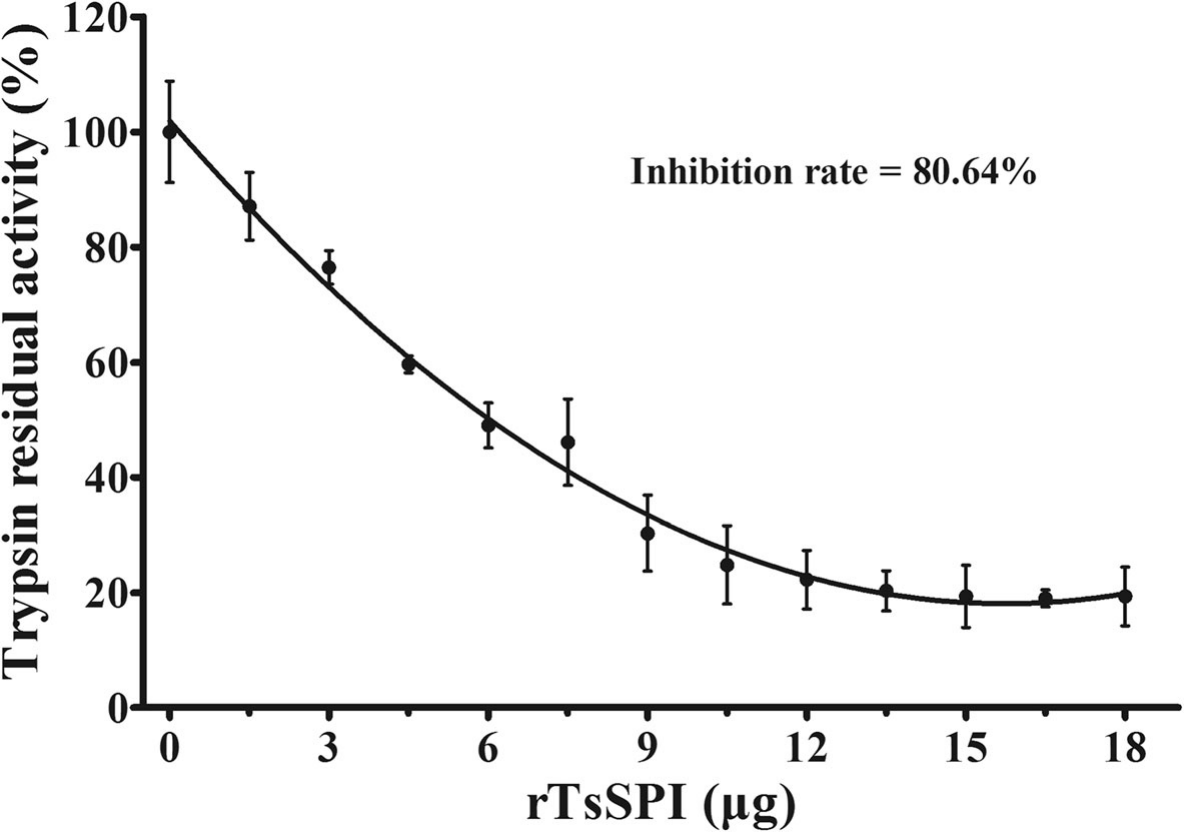
The titration curve graph of trypsin activity inhibited by rTsSPI. rTsSPI of different quantity was added to a fixed quantity of trypsin (1.25 μg). The absorbance at 253 nm was measured by spectrophotometry with the substrate BAEE, and the inhibition rate of trypsin enzymatic activity was calculated as described in method section. Each point is the mean of triplicates

The rTsSPI inhibitory activity on trypsin was determined at various concentrations of the substrate BAEE. The results revealed that the trypsin enzymatic activity was inhibited with rTsSPI. The inhibition rate was 80.64% and the inhibition was rTsSPI dose-dependent (*r*_(12)_ = 0.990, *P* < 0.001) (Fig. 2).

### Stability of rTsSPI activity for inhibiting trypsin

**Fig. 3:**
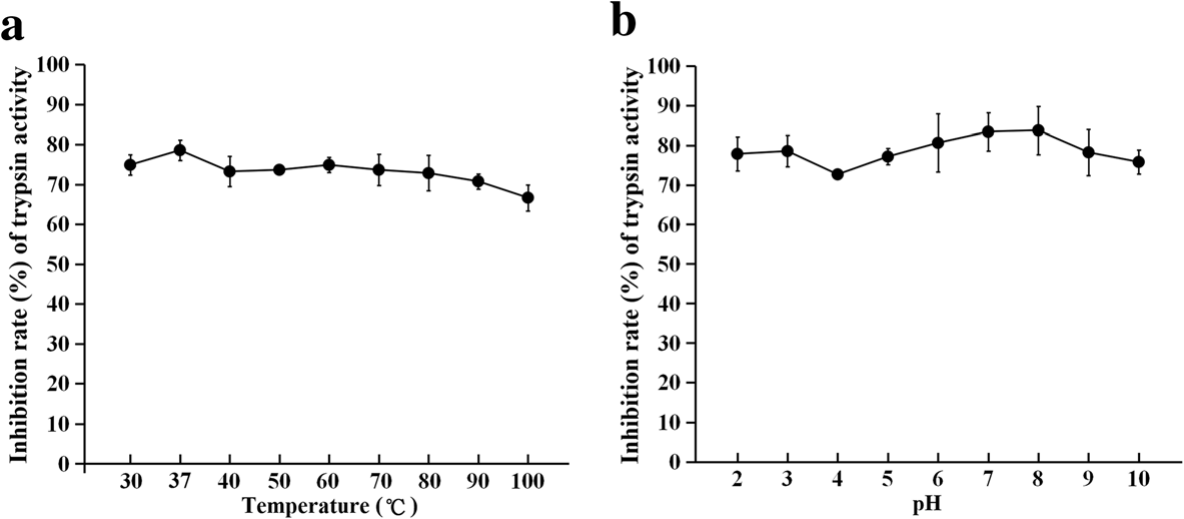
Stability of rTsSPI activity for inhibiting trypsin after incubation at 37 °C for 30 min. **a** Stability of rTsSPI inhibiting activity at different temperature. **b** Stability of rTsSPI inhibiting activity at different pH. The residual enzymatic activity of trypsin was determined with BAEE as a substrate. The experiment was performed in triplicate and the data are the mean ± SD of three tests

The results of temperature effects on rTsSPI inhibitory activity revealed that the inhibitory rate of rTsSPI on trypsin was 80.97% at 37 °C, and exhibited a trend of decrease with temperature increase decreasing to 66.67% at 100 °C (Fig. 3a) thus indicating that TsSPI could maintain its inhibitory activity within a temperature range of 37–100 °C. Pre-incubation of the rTsSPI at pH 2.0–10.0 for 30 min did not affect noticeably the inhibitory effect of rTsSPI on trypsin (Fig. 3b).

### Far Western analysis of rTsSPI binding to IECs

**Fig. 4:**
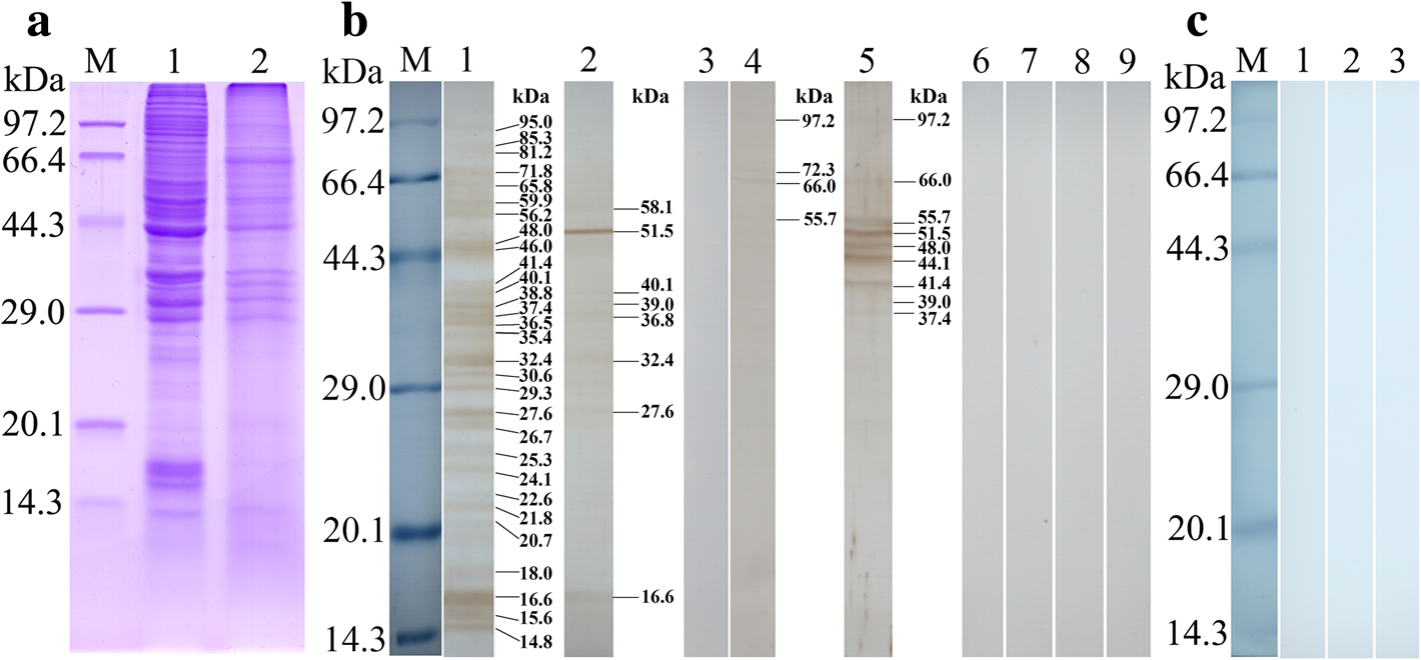
Far Western analysis of rTsSPI binding to IEC proteins. The IEC proteins were analyzed by SDS-PAGE, subsequently the IEC protein binding with rTsSPI was detected in a Far Western analysis. **a** SDS-PAGE analysis of IEC proteins. Lane M: protein marker; Lane 1: IEC lysates; Lane 2: C2C12 lysates. **b** Far-Western analysis of IEC protein binding to rTsSPI. The IEC protein was first incubated using rTsSPI (Lanes 1–3), IL1 ES proteins (Lanes 4–6) or BSA (Lanes 7–9), subsequently recognized by anti-rTsSPI serum (Lanes 1, 4 and 7), infection serum (Lanes 2, 5 and 8), and pre-immune normal serum (Lanes 3, 6 and 9). **c** Far Western analysis of C2C12 protein binding to rTsSPI. The C2C12 protein (Lanes 1–3) was first incubated with rTsSPI, and subsequently incubated with anti-rTsSPI serum (Lane 1), infection serum (Lane 2) or pre-immune serum (Lane 3). There was no binding between rTsSPI and the C2C12 protein

The IEC lysates were analyzed using SDS-PAGE (Fig. 4a). The results showed about 29 protein bands with a molecular weight of 14.8–95 kDa in IEC lysates. Far Western analysis revealed that after incubation with the rTsSPI, all these protein bands from IEC lysates were recognized by anti-rTsSPI serum; of these, about 8 bands (16.6–58.1 kDa) were recognized by the infection serum, but no protein bands were recognized by the pre-immune serum (Fig. 4b). After IEC proteins were incubated with the IL1 ES antigens, anti-rTsSPI serum recognized 4 bands (97.2, 72.3, 66.0 and 55.7 kDa) of IEC proteins, infection serum recognized about 9 bands (37.7–97.2 kDa), and pre-immune serum did not recognize any bands of IEC proteins. No binding between C2C12 proteins and rTsSPI was observed by either anti-rTsSPI serum or infection serum. The results indicated that there was a specific binding and interaction between IEC and rTsSPI.

### IIF analysis of binding of rTsSPI with IEC

**Fig. 5:**
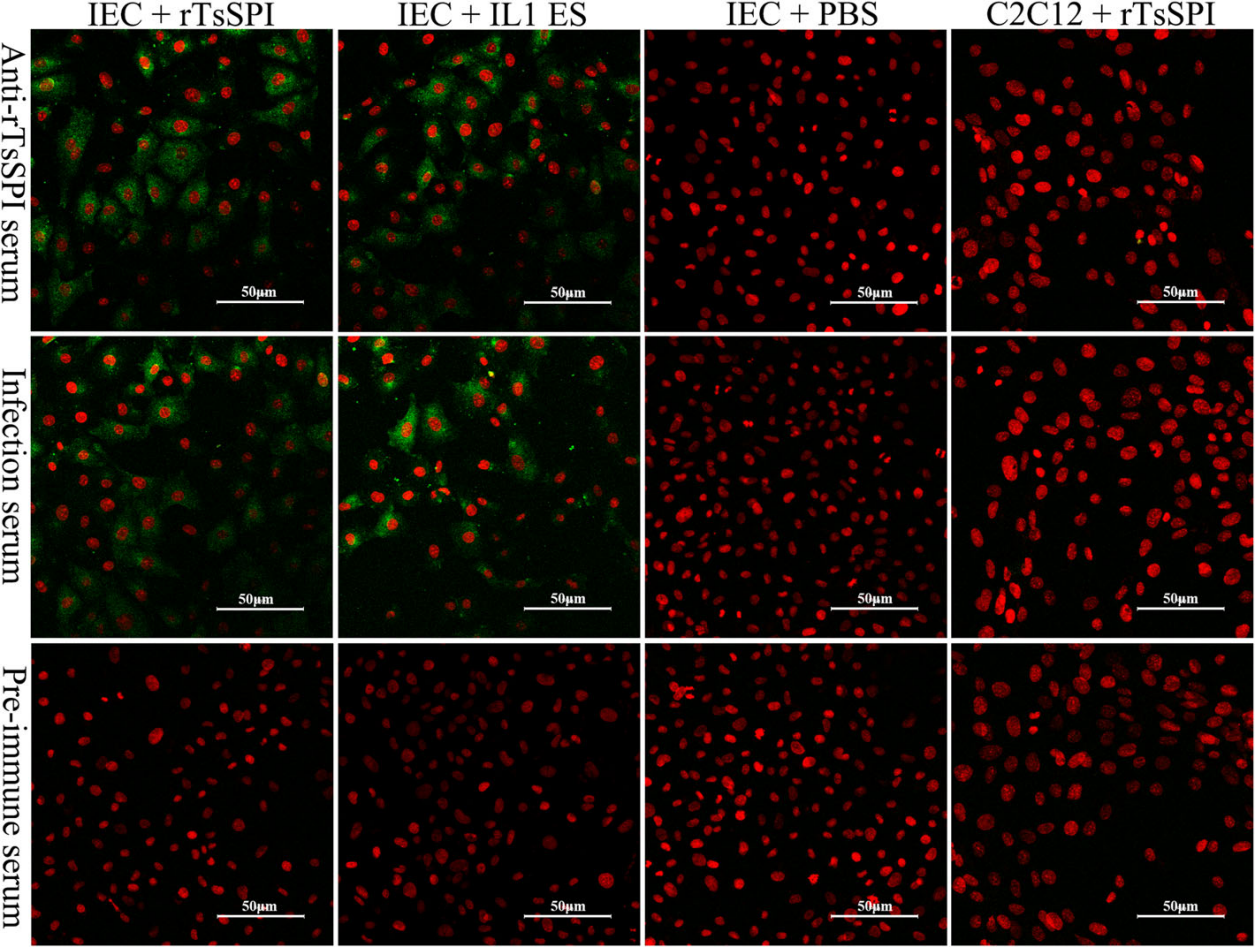
IIF analysis of binding between rTsSPI and IECs (× 200). rTsSPI, IL1 ES antigens or PBS were used for pre-incubating with IEC for 2 h at 37 °C. rTsSPI was also pre-incubated with C2C12 for 2 h at 37 °C. After washes, the pre-incubated IEC and C2C12 was probed by anti-rTsSPI serum, infection serum or pre-immune serum, subsequently colored with goat anti-mouse IgG-FITC conjugate. Propidium iodide (PI) dyed cell nuclei in red

**Fig. 6:**
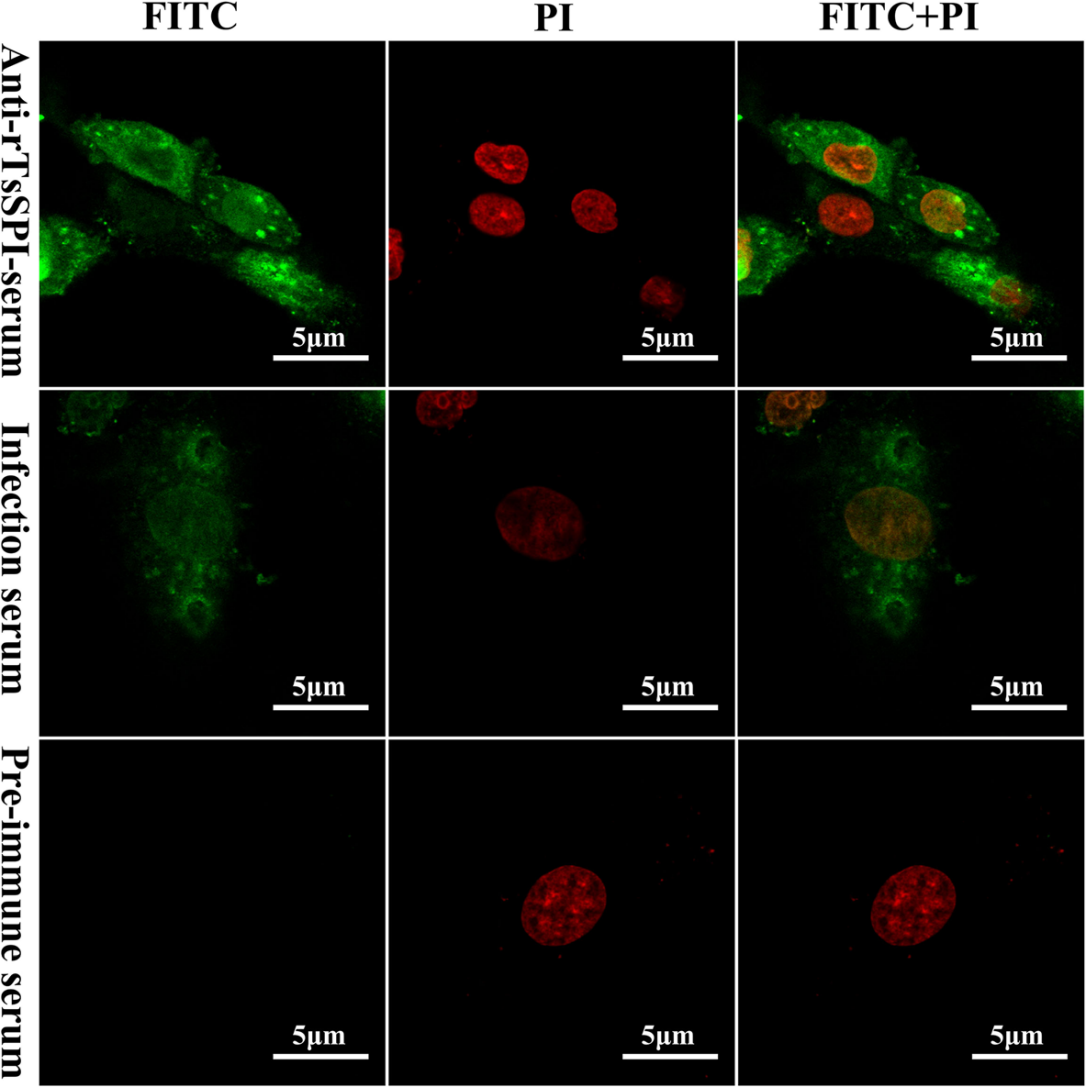
Cellular localization of rTsSPI binding to IEC by confocal microscopy (× 1000). The IEC was pre-incubated by rTsSPI, and then by anti-rTsSPI serum, infection serum or pre-immune serum, and stained using anti-mouse IgG-FITC conjugate. Propidium iodide (PI) dyed cell nuclei in red. *Abbreviations*: FITC, fluorescein isothiocyanate; PI, propidium iodide

After the IEC was pre-incubated with rTsSPI or IL1 ES proteins, immunostaining was observed on IEC surface probed by anti-rTsSPI serum or infection serum, not by pre-immune serum (Fig. 5). Nevertheless, no fluorescent staining on C2C12 pre-incubated with rTsSPI was found by either anti-rTsSPI serum or infection serum. When the positive staining IECs were examined by confocal microscopy, the staining was located on IEC cytomembrane and cytoplasm, indicating that rTsSPI could specifically bind to the IEC membrane and pass into cytoplasm (Fig. 6).

### Specific binding of rTsSPI with intestinal epithelium detected by IIF

**Fig. 7:**
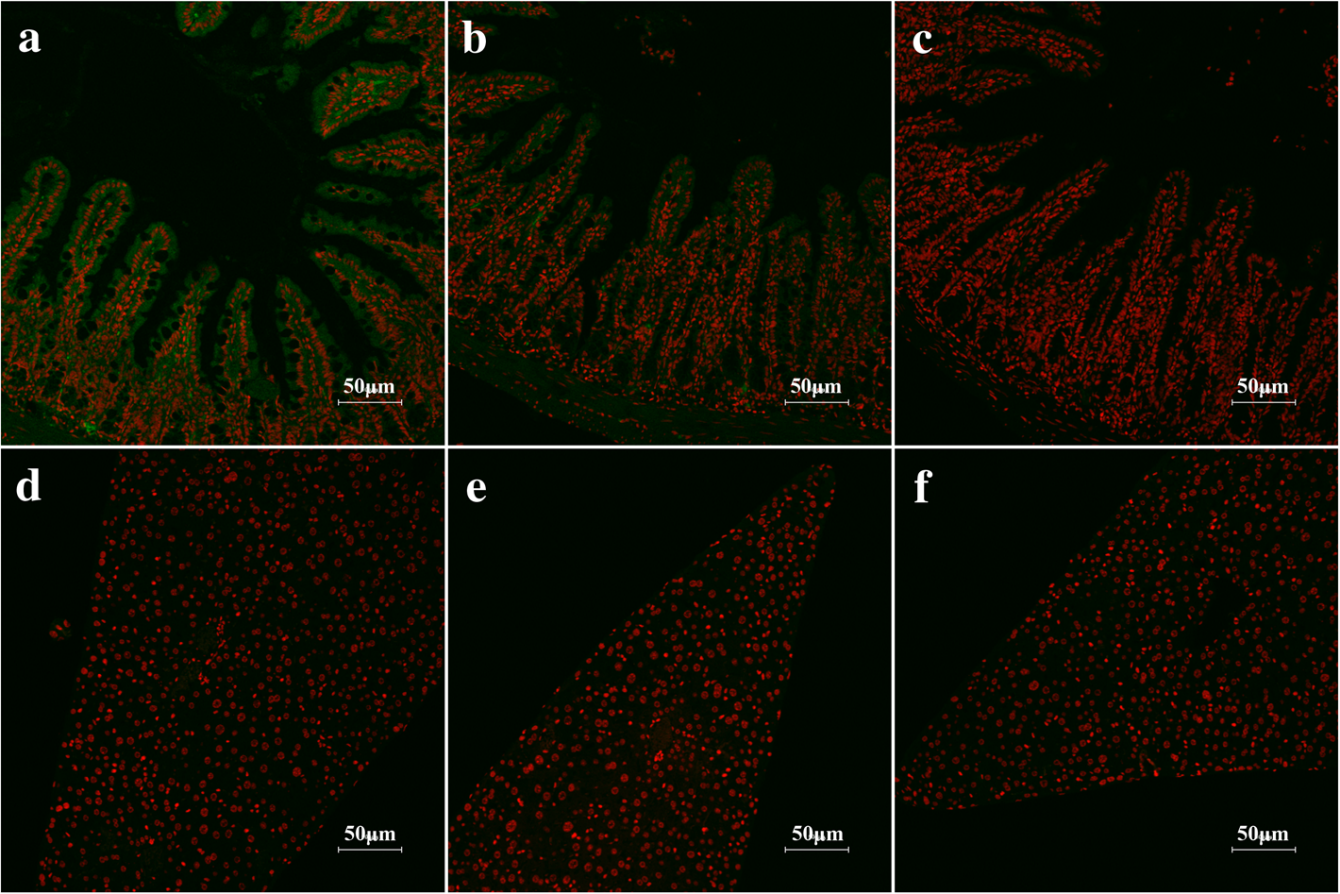
IIF analysis of rTsSPI binding with mouse intestinal epithelium (100×). Tissue sections of the intestines (**a**-**c**) and livers (**e**-**f**) from uninfected mice were incubated with rTsSPI for 2 h at 37 °C. After washing, the sections were probed for 1 h at 37 °C with anti-rTsSPI serum (**a**, **d**), infection serum (**b**, **e**) or pre-immune serum (**c**, **f**), and then with anti-mouse IgG-FITC conjugate. Propidium iodide (PI) dyed cell nuclei in red. These sections were examined by fluorescent microscopy

The results of IIF with intestinal and liver sections revealed that after incubation with rTsSPI, immunostaining on intestinal epithelium was detected by using anti-rTsSPI serum; weak staining was also detected with infection serum (Fig. 7) but no staining was observed with pre-immune serum. Moreover, no fluorescent staining in liver tissues incubated with rTsSPI was detected by either anti-rTsSPI serum or infection serum.

### Inhibiting effects of anti-rTsSPI serum on the IEC invasion by *T. spiralis*

**Fig. 8:**
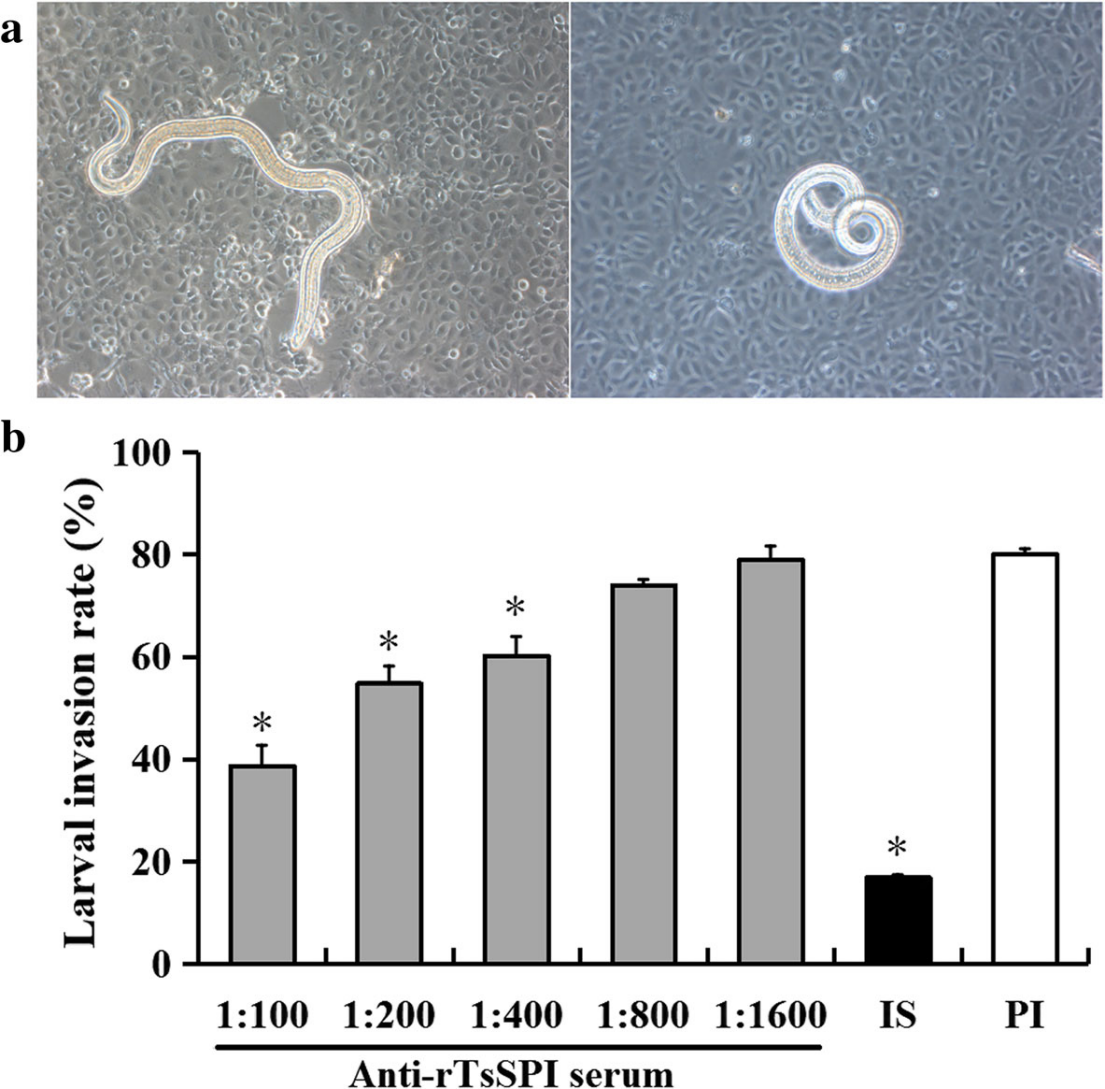
The *in vitro* inhibition of *T. spiralis* invasion of IEC by anti-rTsSPI serum. **a** When IEC monolayer was covered and cultured with the semisolid media containing the larvae at 37 °C for 2 h, the invaded (left) and non-invaded larvae (right) in the IEC monolayer (× 200). **b** Inhibition of larval invasion of IEC by different dilutions of anti-rTsSPI serum. The 1:100 dilutions of infection serum (IS) and pre-immune serum (PI) were utilized as control sera. The results are shown as the percent of the larvae invaded the monolayer out of all larvae added into the media. Asterisks indicate statistically significant differences (*P* < 0.001) in comparison with the pre-immune serum group

When the IEC monolayer was covered by semisolid media containing the larvae, and cultured for 2 h, the larvae invaded and migrated in the IEC monolayer (Fig. 8a). When 1:100 dilution of anti-rTsSPI serum, infection serum and pre-immune serum were added into the media and cultured for 2 h, the invaded larvae in the monolayer represented 38.7%, 16.85% and 80.08%, respectively, of the three groups of sera the difference of larval invasion rate was statistically significant (*χ*^2^_(2)_ = 134.354, *P* < 0.001). The inhibiting effect of the anti-rTsSPI serum on the monolayer invasion was more obvious than those of the pre-immune serum (*χ*^2^_(1)_ = 260.000, *P <* 0.001), and the inhibition was anti-rTsSPI antibody dose-dependent and exhibited a reducing trend with serum dilution elevating (*F*_(4, 11)_ = 160.236, *P* < 0.001) (Fig. 8b) Nonetheless, no apparent inhibition by the pre-immune serum on larval invasion was observed.

### ADCC mediated the killing of NBL

**Fig. 9:**
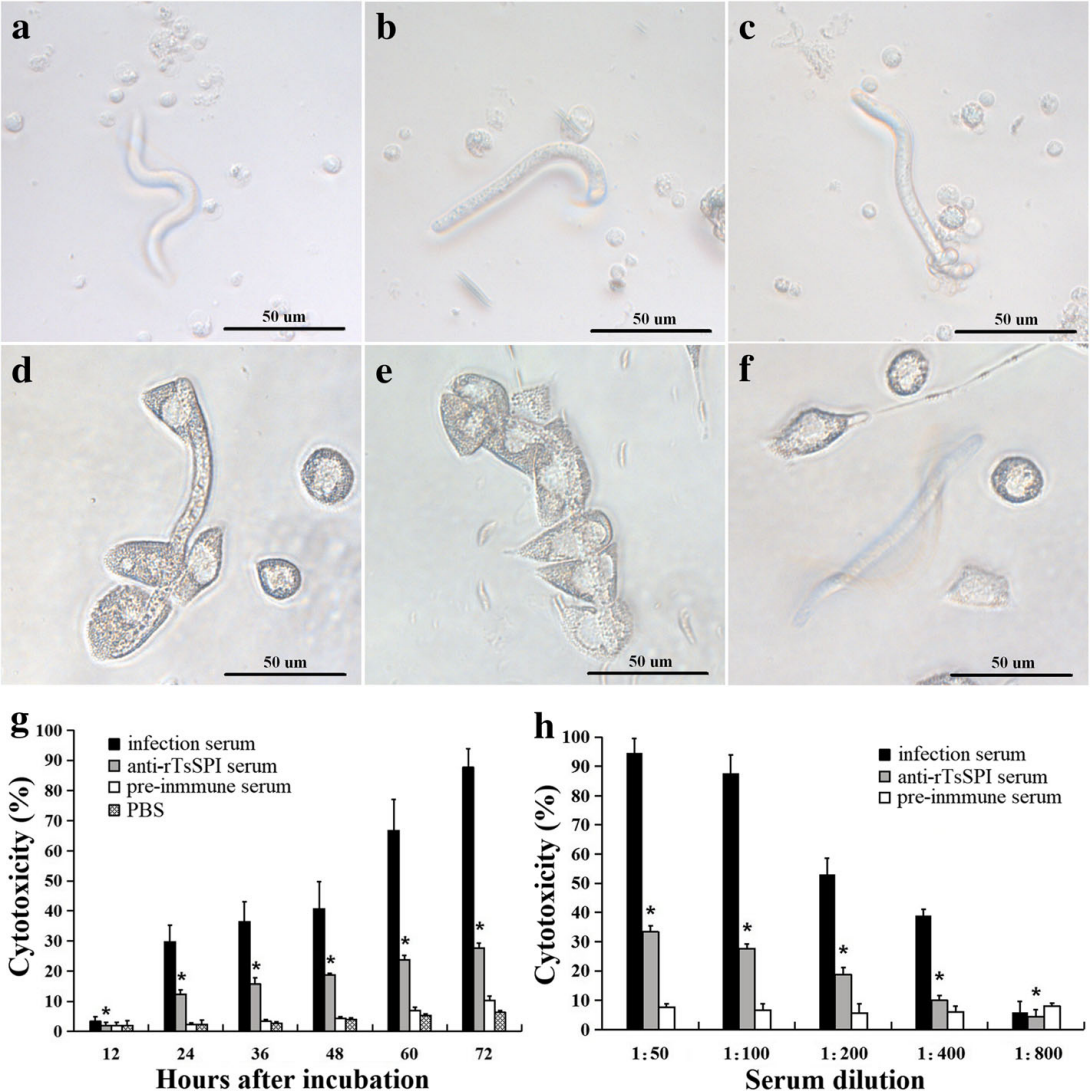
ADCC killing *T. spiralis* NBL. **a-f** Morphology of *T. spiralis* NBL recovered after ADCC test with different cultivation time periods. The NBL were cultured using anti-rTsSPI serum and 1 × 10^5^ mouse peritoneal exudate cells (PECs) at 37 °C for different time periods. **a**, **b** No PECs were adhered to NBL 12 h and 24 h of cultivation and the NBL was wriggling. **c** A few of PECs adhering to NBL 48 h of cultivation and the NBL was limp with weak activity. **d** A dead NBL overlaid with PECs 72 h of cultivation. Infection serum (**e**) and pre-immune serum (**f**) were utilized as control sera. **g-h** ADCC killing *T. spiralis* NBL is anti-rTsSPI antibody dose-dependent and associated with culture time. **g** The NBL were incubated with same dilution (1:100) of various sera for 12–72 h of incubation; the cytotoxicity had an increasing trend with the culture time prolongation. **h** The NBL were incubated with different sera diluted at 1:50 to 1:800 for 72 h; the cytotoxicity exhibited an anti-rTsSPI antibody dose-dependent pattern. Asterisks indicate that the cytotoxicity of the anti-rTsSPI serum exhibited a significant difference (*P* = 0.005) compared with that of the pre-immune serum

The ADCC test revealed that after incubation, anti-rTsSPI serum could mediate the adhering and killing of the PECs to the NBL (Fig. 9). When anti-rTsSPI serum (1:100 dilution) was incubated for 72 h with the NBL and PECs, the ADCC resulted in a visible death of NBL (27.67% cytotoxicity), in comparison to the NBL incubated with pre-immune serum (10.33%, *χ*^2^_(2)_ = 10.526, *P* = 0.01). The cytotoxicity also had an obvious correlation with culture time (*r*_(5)_ = 0.968, *P* < 0.001), and exhibited an increasing trend with extension of culture time (*F*_(5, 13)_ = 117.584, *P* < 0.001). The cytotoxicity was anti-rTsSPI antibody dose-dependent (*r*_(5)_ = 0.984, *P* < 0.001), and had a decreasing trend following the increase of serum dilutions (*F*_(4, 11)_ = 96.813, *P <* 0.001) (Fig. 9).

## Discussion

In this study, the TsSPI from *T. spiralis* adult worms was cloned and expressed. SDS-PAGE analysis and spectrophotometry showed that the rTsSPI reacted strongly with porcine trypsin, had inhibitory activities against porcine trypsin, and the inhibition was rTsSPI dose-dependent, demonstrating that trypsin may be the possible target of the inhibitor [16]. The TsSPI could maintain its inhibitory activity against trypsin within a temperature range of 37–100 °C, and pH values of 2.0–10.0 did not affect noticeably the inhibition effect of rTsSPI on trypsin. However, the precise role that the inhibitor might play in *T. spiralis* invasion and survival in host’s intestine is not completely clear. A hookworm *Ancylostoma duodenale* adult serpin (AduTIL-1) has been expressed, being distributed in cuticle surface, oesophagus and intestines of adult worms; this rAduTIL-1 was found to show inhibitory activity against human neutrophil elastase and pancreatic trypsin. AduTIL-1 may participate in *Ancylostoma* survival in host by targeting relative digestive enzymes and elastase [52]. A *Schistosoma haematobium* serpin was located on the surface of this schistosome and can interact with host cells and proteases [53]. Previous studies showed that the TsSPI was detected in excretory/secretory (ES) proteins of *Trichinella spiralis* AW, widely distributed on the external surface of AW and NBL cuticle [19]. A *T. pseudospiralis* serpin is an exocrine protein and may play a role in the immunoregulation of *T. pseudospiralis* infection by directly acting on host’s cells or humoral molecules [54]. The TsSPI might inactivate the enzymatic activity by forming a complex with host serine protease, which overlays the surface of *Trichinella*, and hence protects the worms from the host’s serine proteolysis. The protection mechanism could be similar to the one described for *Ascaris suum* [55].

We identified the protein-protein interactions between rTsSPI and IECs in this study. Far Western analysis revealed that approximate 24 bands of IEC proteins pre-incubated using rTsSPI were identified by the anti-rTsSPI serum. IIF indicated the rTsSPI specifically bound to intestinal epithelium and IECs, and the location of the binding sites in the IEC membrane and cytoplasm was demonstrated under confocal microscopy. The results indicated that there was an interaction between TsSPI and IECs, and TsSPI might act a major role in parasite invasion of the intestinal epithelium [56]. However, the mechanism of TsSPI and IECs interaction needs to be further studied.

Anti-TsSPI antibodies could inhibit the anti-proteolytic activity of serpins in a way similar to that observed for serpins originating from other parasites, such as *S. japonicum* [57]. Anti-*Trichinella* antibodies could bind to *T. spiralis* surface and form immune complex in the anterior part, which may physically block the worm’s recognition and invasion of intestinal mucosa [31, 58]. Our results showed that anti-rTsSPI antibodies could significantly inhibit the larval invasion of IECs and the inhibition was anti-TsSPI antibody dose-dependent. When infection serum was used in the invasion experiment, the inhibition of parasite invasion by infection serum was more obvious than that of the anti-rTsSPI serum. This may be due to the fact that the antibodies to other invasion-related proteins of *T. spiralis* (e.g. glutathione S-transferase, cysteine protease) in infection serum were also involved in inhibition of the invasion [45, 47, 59, 60]. Previous studies showed the serpins were synthesized mainly in the early developmental stages of the parasites. *Schistosoma japonicum* serpin is a tegumental protein, and expressed only at cercarial and adult stages; vaccination with the rSj serpin triggered high levels of specific antibody responses in vaccinated mice and showed partial protection against challenges [57]. The serpin content of *Brugia malayi* infective stage larvae was 10–16 times higher than those of adults or microfilariae [61]. Our results suggested that TsSPI is an early invasion-related protein in the process of *T. spiralis* infection.

Previous studies demonstrated that ADCC-mediated destruction of *T. spiralis* NBL was dependent on specific anti-*Trichinella* IgG [62, 63]. Cytotoxicity against NBL in the lungs of *T. spiralis-*infected mice was found to be IgE-mediated by Falduto et al. [64]. To investigate the cytotoxicity of anti-rTsSPI antibodies, an *in vitro* ADCC test was also performed in the present study. Our results revealed that anti-rTsSPI antibodies took part in NBL sacrificing. The PECs attached to and destroyed the NBL under the mediation of anti-rTsSPI serum and the destruction was anti-rTsSPI antibody dose dependent. These results demonstrated that TsSPI might participate in *Trichinella* invasion of the host and it is likely a potential vaccine target molecule against enteral stage worms of *T. spiralis* infection.

## Conclusions

The rTsSPI had the inhibitory activities against porcine trypsin. The rTsSPI especially bound and interacted with host’s intestinal epithelium and IECs and the binding sites were located in IEC membrane and cytoplasm. Anti-rTsSPI antibodies suppressed the *T. spiralis* invasion of host’s IECs in a dose-dependent mode. The TsSPI-specific antibodies also participated in the destruction of *T. spiralis* NBL *via* an ADCC-mediated manner. The results demonstrated that TsSPI might participate in the invasion of this nematode in the host, and is likely a potential vaccine target against *T. spiralis* enteral stages.

## Abbreviations

ADCC: Antibody-dependent cellular cytotoxicity
AW: Adult worms
BAEE: N-Benzoyl-DL-arginine ethyl ester hydrochloride
BSA: Bovine serum albumin
DAPI: 4’,6-diamidino-2-phenylindole
dpi: Days post-infection
ES: Excretory-secretory
FITC: Fluorescein isothiocyanate
IECs: Intestinal epithelial cells
IIF: Indirect immunofluorescence
IL1: Intestinal first-stage infective larvae
ML: Muscle larvae
NBL: Newborn larvae
PBST: PBS-0.5% Tween 20
PEC: Peritoneal exudates cells
PI: Propidium iodide
qPCR: Quantitative PCR
RCL: Reactive central loop
SD: Standard deviation
serpin: Serine protease inhibitor
TsSPI: *T. spiralis* serine protease inhibitor
